# A Rare Case of Serous Papillary Ovarian Epithelial Type Carcinoma in Testis: A Rare Testicular Neoplasm 

**Published:** 2019-06

**Authors:** Alireza Abdollahi, Fereshteh Ensani, Mohsen Ayati, Sepideh Jahanian, Arezoo Eftekhar Javadi, Atieh Khorsand

**Affiliations:** 1 Department of Pathology, Imam Khomeini Hospital Complex, School of Medicine, Tehran University of Medical Sciences, Tehran, Iran; 2 Department of Urology, Imam Khomeini Hospital Complex, School of Medicine, Tehran University of Medical Sciences, Tehran, Iran; 3 Student Research Committee, School of Medicine, Iran University of Medical Sciences, Tehran, Iran; 4 Department of Pathology, Sina Hospital, School of Medicine, Tehran University of Medical Sciences, Tehran, Iran; 5 Department of Pathology, Shariati Hospital Complex, School of Medicine, Tehran University of Medical Sciences, Tehran, Iran

**Keywords:** Serous Papillary, Ovarian Carcinoma, Testicular Neoplasm

## Abstract

Ovarian epithelial type carcinomas of testis are an extremely rare group of tumors, a few cases of which having been reported. We present the case of a 67-year-old man, presented with testicular mass and inflation, who underwent radical orchiectomy and pathological and immunohistochemical assessments revealed serous papillary carcinoma of ovarian epithelial type tumor of testis.

## Introduction

Ovarian epithelial type carcinomas of testis are remarkably rare and hard to diagnose histologically as they are identical to the epithelial tumors of ovary and similar to the mesothelioma of tunica vaginalis testis (1).

Histologically these tumors are divided into the following patterns: serous, mucinous, endometrioid, clear, transitional (Brenner) and squamous (1).

The origin of these tumors is still debatable but is more likely to be from the persisting remnants of Müllerian ducts in the male testicular or paratesticular regions (2).

The immunohistochemical markers have reported to be positive for CA125, Cytokeratin 7, CD 15, epithelial membrane antigen and Ber-EP4 (2, 3).

Although these markers are not very specific they help rule out other differential diagnoses like malignant mesothelioma (3, 4).

As only a few cases of this entity have been reported in the English literature, we report the case of a serous papillary carcinoma of ovarian epithelial type in the testes with the aim of recalling that diagnosis of this rare complication should be taken note of when evaluating testicular masses in males.

## Case report

The patient was a 67-year-old Iranian male who had undergone a repair hydrocele surgery in 2014 following a chief complaint of an inflamed left testicle. The pathology of the surgically removed sample reported inflamed fibrous wall compatible with the clinical diagnosis of hydrocele.

The patient returned with a relapse of the mass at the same spot in 2015. Sonography showed a solid heterogenic mass in the superior part of the left testicle. The patient underwent left radical orchiectomy. The pathologic examination of the specimen was consistent with papillary serous carcinoma, which invaded the paratesticular structures and vasculature and extended through tunica albuginea with involvement of tunica vaginalis. With respect to this diagnosis the patient also underwent 4 chemotherapy and 25 radiotherapy sessions.

Several months later in the summer of 2016, the patient presented with inflated left inguinal region and a palpable mass, which was reported by an ultrasonography as a mass measuring 12 millimeters in diameter. He was put on antibiotic therapy for two weeks as his symptoms raised suspicion of infection. As the inflation did not regress, spiral CT-scan was conducted which showed lymph node invasion and lymphadenopathy of the para-aortic region with the largest one measuring 43 millimeters in diameter. No abdominal and pelvic fluid was observed.

The patient’s past medical history was significant for diabetes and a past surgical history of cholecystectomy and CABG.

The familial history of malignancies was negative.

The patient’s lab data are as follows:

Hemoglobin 11.1 gr/dl, Platelet 237000, SGPT: 15 U/L (normal range < 37), SGOT: 31 U/L (normal range < 41), Alk-p: 404 U/L (normal range 80-306 U/L), LDH: 505 U/L (normal range < 480 U/L), CEA: 0.74 ng/ml (normal range < 5 ng/ml), CA-125: 6.30 U/mL (normal range < 35 U/ml), AFP: 6.1 ng/ml (normal range < 10 ng/ml), Beta-HCG < 2 mlu/ml (normal range < 5 mlu/ml)

The patient’s excised tumor following orchiectomy was examined by the pathologist and reported as a papillary serous carcinoma of testis with the greatest diameter being 11.5 cm, which invaded the paratesticular structures and extended through tunica albuginea with the involvement of tunica vaginalis (pT2).

Vascular invasion was present. No intra-tubular germ cell neoplasia was seen.

**Figure 1:**
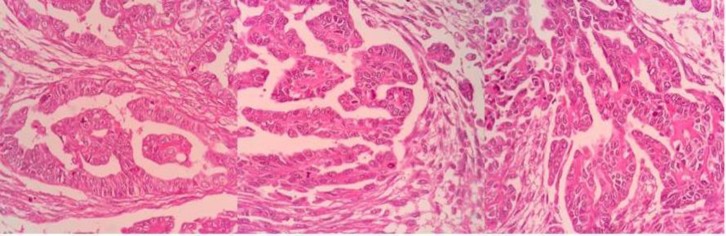
A neoplastic tissue composed of proliferated cuboidal to low-columnar cells arranged in glandular and tubule-papillary structures. Variable degrees of nuclear atypia, hyalinization of papillary cores also seen

The patient’s slide and block were sent to the pathology of this center for consultation. Microscopic examination showed epithelial neoplasm with tubulopapillary growth pattern consistent with the epithelial ovarian papillary serous carcinoma (Testicular structure around a neoplastic tissue, composed of proliferated cuboidal to low-columnar cells arranged in glandular and tubule-papillary structures. Variable degrees of nuclear atypia, hyalinization of papillary cores also seen. The intervening stromal is fibrotic but there are foci where stromal is cellular) (Figure 1).

**Figure 2:**
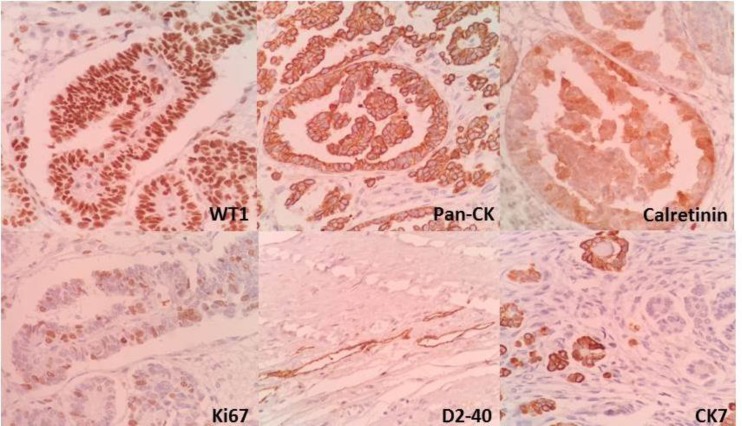
Immunohistochemical assessments of the epithelial cells of the tumor were positive for pan-CK, CK7, Calretinin, WT-1 and D2-40

Immunohistochemical assessments of the epithelial cells of the tumor were positive for pan-CK, CK7 (multifocal), Calretinin(multifocal), WT-1 and D240 (focal). No CK20, CA125 or CEA were detected. Proliferative activity Ki67 was also significant (Figure 2).

## Discussion

Regarding the 2016 WHO classification of tumors of testis, ovarian epithelial type tumors are classified in the group of miscellaneous tumors of testis (4).

These tumors are more commonly paratesticular rather than testicular (2, 5).

The origin of the tumor is still under debate but some theories suggest the origin of Müllerian duct remnants in testis (5). It could also be originating from epididymis, spermatic cord or mesotheliomafollowing Müllerian metaplasia of tunica vaginalis testis.as they are observed and reported in microscopic examinations in several cases (6) Intratesticular tumors are more likely to originate from mesothelioma (2).

Histologically they are classified as serous, mucinous, endometrioid, clear cell, Brenner (transitional), ordered based on their frequency respectively (2).

These tumors are found in patients within the age group of 14-68 years (4).

The mean age for borderline serous tumors is 56 and for invasive neoplasms is 31 years (2).

Patients generally present with scrotal swelling, hydrocele, dull pain or scrotal mass (6, 7).

Although, radiological findings and clinical presentations are not able to distinguish between this tumor and other similar neoplasms, clinical history and physical examination along with laboratory assessments such as blood chemistry and tumor markers, Ultrasonography of the testes, CT scan and chest X-ray should be taken before the surgery (5).

Since the diagnosis of ovarian epithelial type carcinomas is difficult based on the morphological similarities(5), other diagnoses such as malignant mesothelioma of the tunica vaginalis of testis should also be considered, as these tumors may be microscopically similar to ovarian type epithelial tumors and they can be ruled out with the help of immunohistochemically markers like calretinin (3,7).

Other differential diagnoses include other ovarian type epithelial tumors, adenocarcinoma of the rete testis, adenocarcinoma of the epididymis and other metastatic carcinomas (1, 5).

Microscopically serous ovarian type neoplasmsdemonstrate papillae with fibrovascular cores and a border of stratified cuboidal or columnar epithelium with different mitotic activity (8,9). Carcinomas exhibit invasion and are usually non-cystic while borderline tumors are usually cystic and have a thin fibrous capsule. The presence of psammoma bodies, ciliated and hobnail cells could help with the diagnosis of serous neoplasms (2).

This is while malignant mesotheliomas lack psammoma bodies and exhibit low cellularity (3).

Metastatic lesionsfrom lungs, gastrointestinal tract, bladder and prostate, which could also demonstrate papillary features are ruled out based on clinical presentations and radiographies as these lesions are usually multiple, bilateral and exhibit vascular and lymphatic invasion with an interstitial growth pattern. Adenocarcinoma of the epididymis is consisting of tubular patterns with destruction of epididymis (10-12).

The diagnosis of the adenocarcinoma of the rete testis usually requires the exclusion on other diagnoses based on histologic morphologies and immunohistochemicalmarkers (5).

In contrast to the borderline serous tumors which are excellent in case of complete excision, the papillary serous carcinomas are more likely to metastasize and recur (2).

Immunohistochemical markers could also be used as a helpful diagnostic tool and to rule out other diagnoses. Serous tumors express ovarian epithelial tumor markers such as CA125, Cytokeratin 7, CD 15, epithelial membrane antigen and Ber-EP4 (3).

This is while mesotheliomas are usually D2-40, calretinin, WT-1, thrombomodulin and CK 5/6 positive (2,3).

Müllerian papillary serous neoplasms express WT-1 and CA125 (2).

In addition, the marker PAX8 could be useful for differentiating mesothelioma and serous ovarian epithelial type carcinomas as it is rarely expressed in mesotheliomas. WT1 is not a good distinguishable marker as it is expressed in both mesotheliomas and ovarian epithelial type tumors (5).

Other immunohistochemical markers include CEA, Leu-M1, B72.3 and Ber-EP4 for adenocarcinomas, CD10 and calretinin for wolffian-derived structures and inhibin for sex-cord associated tumors (2).Metastatic lesions may exhibit CK7, CK20, TTF1, PSA, HEPAR-1, CDX2, and etc (7).

In a case report conducted by Celdrán and et al. a 44-year-old male was presented by testicular swelling in which the mass was reported as primary mucinouscystadenocarcinoma, which is a rarer pathology relating to ovarian epithelial type carcinoma, and the patient was also at a younger age than our patient (8).

Another case reported by Aravindet. al. was a 67-year-old male patient with high grade ovarian epithelial type serous cystadenocarcinoma of testis which exhibited as extensive metastasis in supra clavicular, mediastinal and abdominopelvic regions. IHC markers of CA-125 and Ki 67 were performed for this case which came back positive (7).

Our patient manifested metastasis after he returned with recurrence. Immunohistochemical assessments in our patient showed positive Pan-CK, CK 7 (multifocal), calretinin(multifocal), WT-1 and D240 (focal). CA-125, CK 20 and CEA were not detected.

## Conclusion

We reported this case to indicate the fact that although these types of tumors are intensely rare in males, they should be kept in mind as they may be invasive and recurring. Immunohistochemistry is a useful tool for diagnosis as microscopic findings may not be exclusive.
